# 5G Key Technologies for Helicopter Aviation Medical Rescue

**DOI:** 10.2196/50355

**Published:** 2024-08-01

**Authors:** Wei Han Sr, Yuanting Li 2nd, Changgen Chen 3rd, Danni Huang, Junchao Wang, Xiang Li, Zhongliang Ji, Qin Li, Zhuang Li

**Affiliations:** 1 Emergency Department Shenzhen University General Hospital Shenzhen China; 2 Guangdong Yitong Software Guangzhou China; 3 Information and Support Division Shenzhen Mindray Biomedical Electronics Co Shenzhen China; 4 Shenzhen branch of Guangdong Co, Ltd China Mobile Communications Group Shenzhen China; 5 School of Microelectronics and Communication Engineering Chongqing University Chongqing China; 6 Technical Department Shenzhen Eastern General Aviation Co, Ltd Shenzhen China

**Keywords:** low airspace, helicopters, medical aid, 5G technology, aeronautical engineering

## Abstract

Rapid global population growth and urbanization have heightened the demand for emergency medical rescue, with helicopter medical rescue emerging as an effective solution. The advent of 5G communication technology, characterized by large bandwidth, low latency, and high reliability, offers substantial promise in enhancing the efficiency and quality of helicopter rescue operations. However, the full integration of 5G technology into helicopter emergency medical services is still in its nascent stages and requires further development. In this viewpoint, we present our experience from the Shenzhen University General Hospital of the application of 5G low-altitude network communication technology, body area network disease sensing technology, and 5G air-ground collaborative rapid diagnosis and treatment technology in aeromedical rescue. We consider that the 5G air-to-ground collaborative rapid diagnosis and treatment technology enables high-quality remote consultation, enhancing emergency medical rescue and providing strong support for future rescue operations.

## Introduction

Helicopter medical rescue has become an effective option for emergency medical assistance, as populations and urbanization increase [1]. Such rescues must quickly and accurately determine the target location, cooperate closely with ground medical personnel, maintain stability during high-speed flight, and achieve real-time data transmission and efficient medical treatment [2,3]. To meet these needs, effective communication technology is essential. As one of the most advanced mobile communication technologies at present, 5G technology has advantages such as large bandwidth, low latency, and high reliability. For medical rescue, 5G has many advantages [4]. Medical experts can guide on-site medical personnel to provide treatment through remote video and other means, thereby improving the efficiency and quality of medical rescue [5,6].

The development of 5G technology started in 2018, and the first 5G network was put into use in South Korea. Since then, 5G technology has rapidly developed globally. At present, many countries, including the United States, Japan, South Korea, and various countries in Europe, have begun commercial deployment of 5G networks. Applications of 5G technology are also being explored. For example, in the United States, 5G technology has been widely applied in fields such as intelligent transportation and intelligent manufacturing. In Europe, 5G technology is applied in industrial automation and robot control [7]. In Asia, South Korea and Japan are exploring 5G technology in intelligent manufacturing and intelligent health care. China has also led the world in the research and applications of 5G technology [8,9]. Since the end of 2019, China has built the world’s largest 5G network, and at present, 31 provinces and cities across the country have opened 5G commercial services. China has applied 5G technology to fields such as intelligent manufacturing, intelligent logistics, and smart cities; while in health care, it has been used for telemedicine and medical image transmission. Companies such as Huawei and ZTE are leading in the research and development of 5G technology. At the same time, the Chinese government is actively promoting its development and has formulated a series of policy measures to support the promotion and applications of 5G technology.

On August 7, 2022, the aviation rescue drill at Shenzhen University General Hospital successfully adopted 5G private network access [10]. We developed aviation medical rescue programs and services through a cooperation agreement with Shenzhen Eastern Navigation Co, Ltd, which provided essential support and guarantees. The work started with 4 aviation medical rescue drills held from September 2019 to December 2022 in order to lay the technical foundation. The types of diseases considered during these drills included multiple injuries to the whole body and spinal cord, and the scenarios included traffic accidents and tunnel scenes. From November 2019 to March 2023, seven aviation medical rescue operations were conducted covering multiple systemic fractures, spinal injuries, drowning pulmonary edema, multiple organ failure, hemopneumothorax, and other diseases. The types of rescue operations were joint mountain search and rescue, seaside drowning rescue, and traffic accident rescue, with a flight distance of 60-600 km and a flight time of 10-136 minutes. These operations involved the first aerial cardiopulmonary resuscitation (CPR), medical staff delivery, and emergency medical treatment. Among the rescue operations, the joint mountain search and rescue used highly complex technologies such as hovering, winches, and aerial lifting, providing significant support for treating patients [11].

This viewpoint paper explores 5G key technologies for helicopter aviation medical rescue to address the shortcomings of traditional air rescue methods in information transmission, positioning accuracy, real-time data transmission, and medical treatment efficiency. We demonstrate the potential of the applications of 5G technology in helicopter medical rescue in terms of improving rescue efficiency, shortening patient treatment time, and enhancing the collaborative combat ability of rescue teams.

## Innovative Technology

### Overview

There are 3 major problems for medical rescue operations in the air: difficulty in communication, difficulty in disease monitoring, and difficulty in collaborative diagnosis and treatment. To overcome these problems, our team put forward a series of important technological innovations such as developing airborne 5G network communication technology and achieving efficient and stable communication. The body area network disease perception technology provided precise real-time monitoring and 5G air-to-ground collaborative rapid diagnosis and treatment technology, which strengthened the collaborative capacity and efficiency of emergency response teams. Hence, these innovations brought unprecedented breakthroughs to the field of aviation medical rescue, significantly improving rescue effectiveness.

### Low Airspace Medical Rescue 5G Network Technology

#### Low-Altitude 2.6+4.9-GHz Collaborative Networking

**Figure 1:**
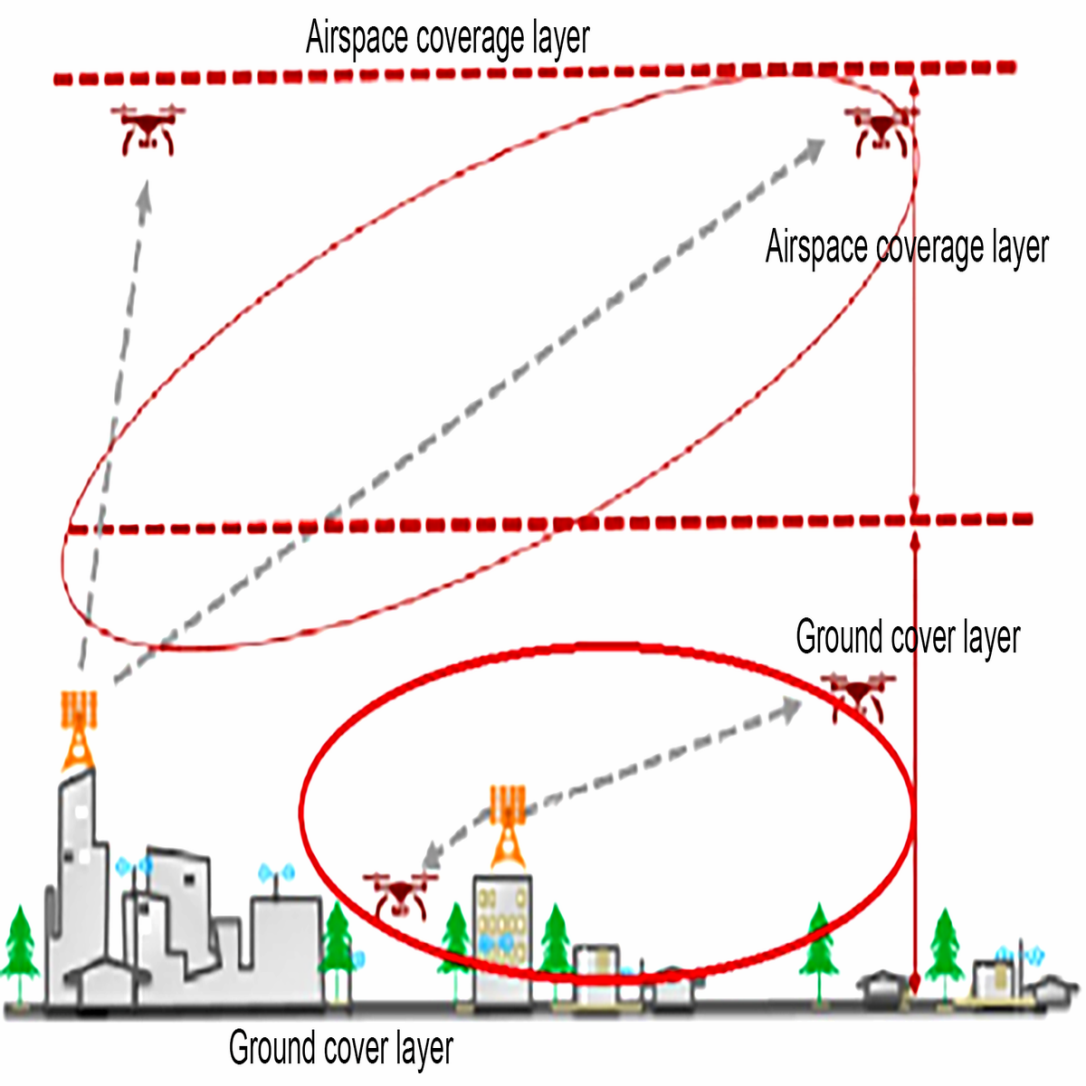
Schematic diagram of 2.6+4.9-GHz dual-frequency collaborative 5G networking.

The low-altitude medical rescue helicopter provided side lobe coverage of a 2.6-GHz 5G down-tilt base station as a 5G private network access signal during takeoff and landing, with a true height less than 150 m. The main or side lobes of a 4.9-GHz 5G up-tilt base station provided 5G private network signal access during takeoff or landing and flight at a true height of 150 m or above. The collaborative coverage of 2 frequency bands of base stations provided seamless switching of 5G private network access for helicopter vertical takeoff and landing and horizontal flight. The network switching delay was ≤30 milliseconds (Figure 1).

#### Adopting 5G+Global Navigation Satellite System External Suction Cup Antenna

**Figure 2:**
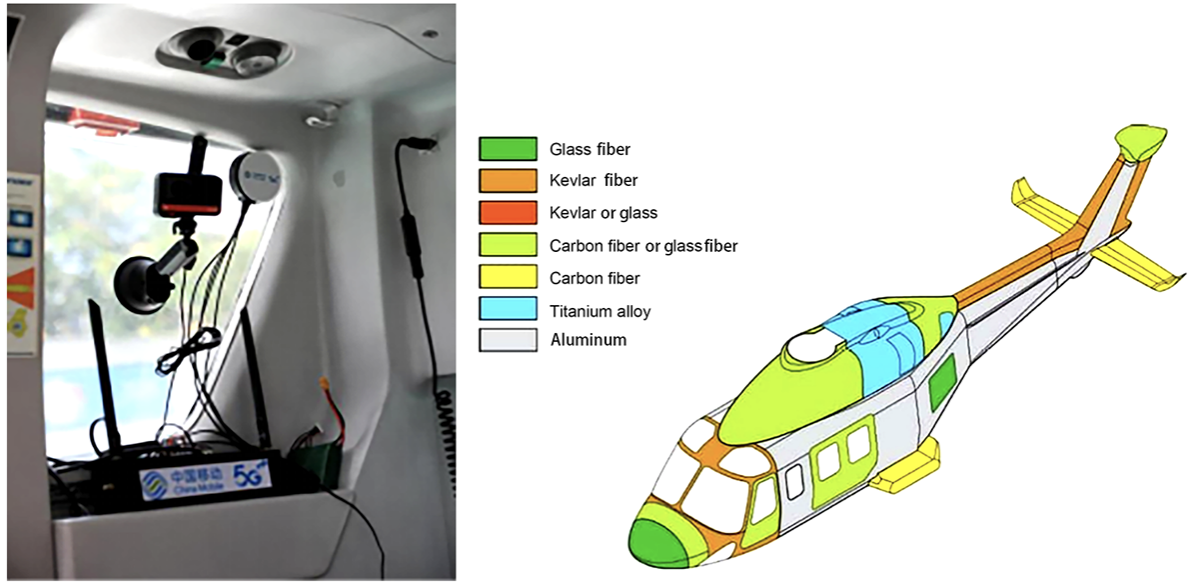
Deployment image of the external 5G+global navigation satellite system antenna (the antenna was derived from the external suction cup antenna of Guanghetong BGS-910).

The aircraft body mainly comprises aluminum, significantly attenuating the signal after blocking radio waves. Therefore, we designed an external pull-away suction cup antenna to dock with the onboard 5G router. The external suction cup antenna could be installed on the glass window side of the helicopter (Figure 2) and directionally receive 5G signals from the external airspace of the aircraft body through glass (window) materials with low signal attenuation.

This installation was made on a domestic medical rescue helicopter, with the novel and successful deployment of 5G-Customer Premises Equipment. After testing, under this installation method, the attenuation difference of 5G signal inside and outside the body was only 8 dB, while 5G-CPE’s built-in rubber rod antenna deployed inside the cabin, and the attenuation of 5G signal by the metal material of the aircraft body was 20 dB, which is a significant difference.

#### Collaboration of 5G+Beidou Positioning Technology

**Figure 3:**
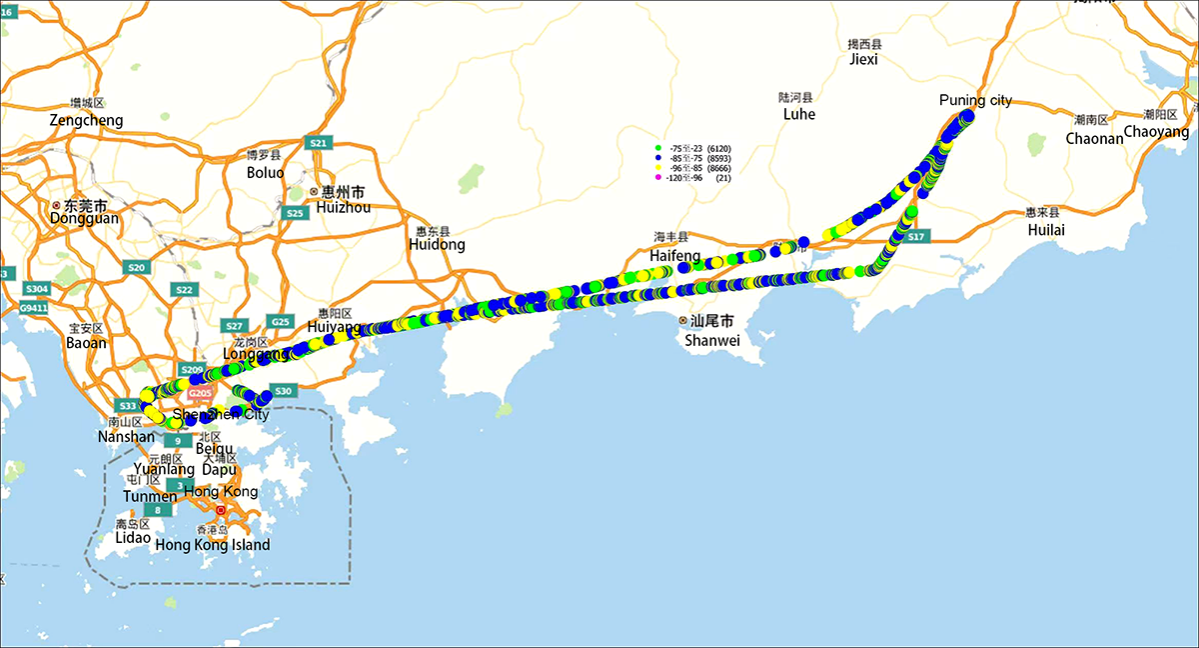
Beidou positioning trajectory map obtained from 5G module.

Next, we examined the Beidou positioning capability provided by the 5G-Customer Premises Equipment module (Figure 3). The data were transmitted back to the emergency platform deployed by the hospital via the 5G private network to locate the real-time flight position of the rescue helicopter.

**Figure 4:**
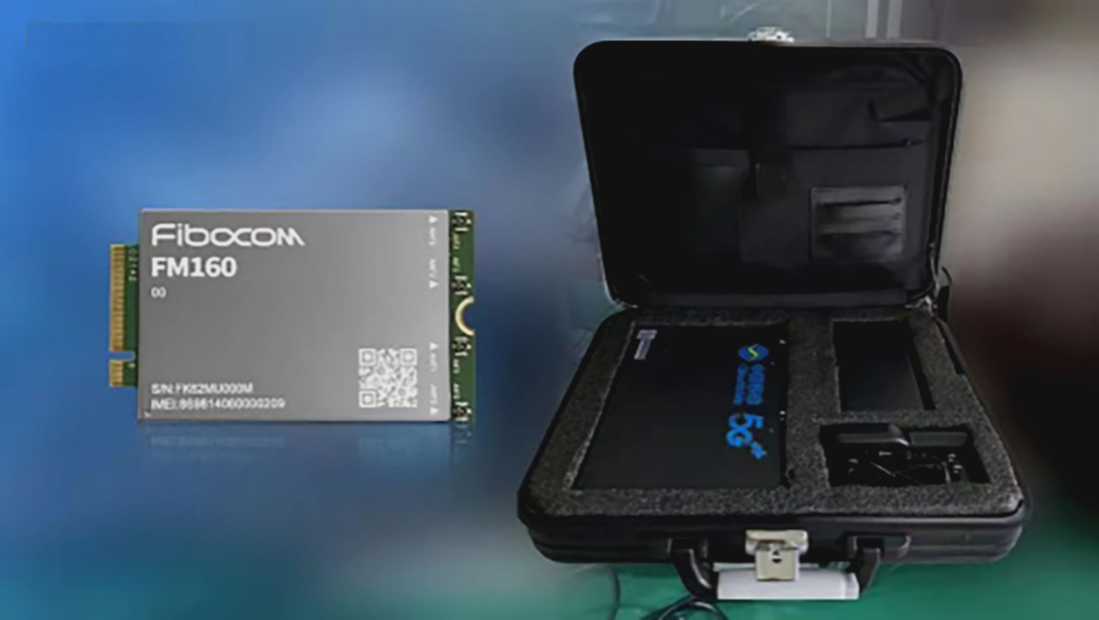
5G module–integrated 5G air rescue equipment with global navigation satellite system capability. The module was derived from Guanghetong FM160.

The traditional positioning scheme for airspace helicopters generally uses a GPS for the helicopter’s built-in flight-positioning module. It does not output positioning data to external systems. It only interfaces with local airspace management departments (regional civil aviation bureaus or air traffic control bureaus) for airspace flight management. For this scheme, we adopted the Guanghetong FM160 model 5G air rescue equipment (Fibocom; Figure 4) and used the global navigation satellite system capability of the 5G module and the antenna to provide global navigation satellite system reception, obtaining real-time positioning information for low-altitude medical rescue, and the information output of real-time positioning of low-altitude medical rescue was realized.

### Air Body Area Network Disease Awareness Technology

#### Construction of a Body Area Network Transmission Scheme Based on Posture Feature and Channel Quality

**Figure 5:**
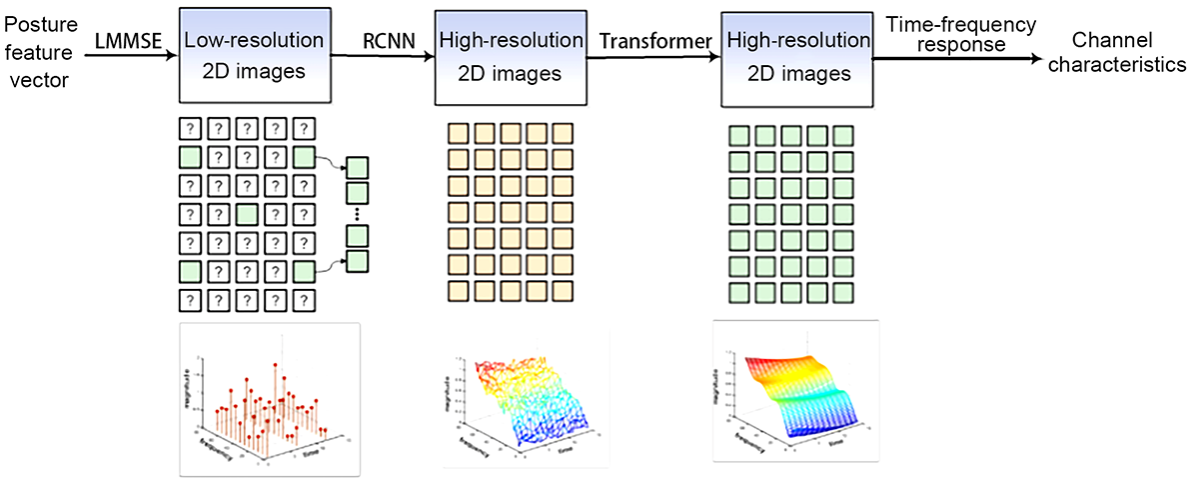
Detection of channel characteristics in body area networks. LMMSE: linear minimum mean square error; RCNN: region-based convolutional neural network.

Communication energy consumption is the key factor that restricts the wider applications of a network with limited energy resources. Human activities drastically affect near-body communication channels, leading to strong time-varying characteristics. This limits the performance of communication energy consumption optimization methods based on wireless sensor networks [12,13]. Therefore, designing an intelligent communication mechanism for body area networks should consider the strong time-varying characteristics of the channel so that the overall transmission energy efficiency can be improved. Our approach was to design a near-body channel estimation algorithm based on deep learning. Treating the time-frequency response of the pilot point as a low-resolution 2D image, using convolutional neural networks and memory networks to generate high-resolution images, and obtaining complete time-frequency response characteristics helped predict and evaluate the current channel quality (Figure 5). Further, we combined human dynamic channel quality estimation and prediction procedures and studied energy-efficient and adaptive access protocols and resource allocation schemes for improved service quality to achieve stable, flexible, and low-power transmission effects.

#### Proposing a Volume Domain Network Data Encryption Method Based on Physical Signal Features and Chaotic Mapping

**Figure 6:**
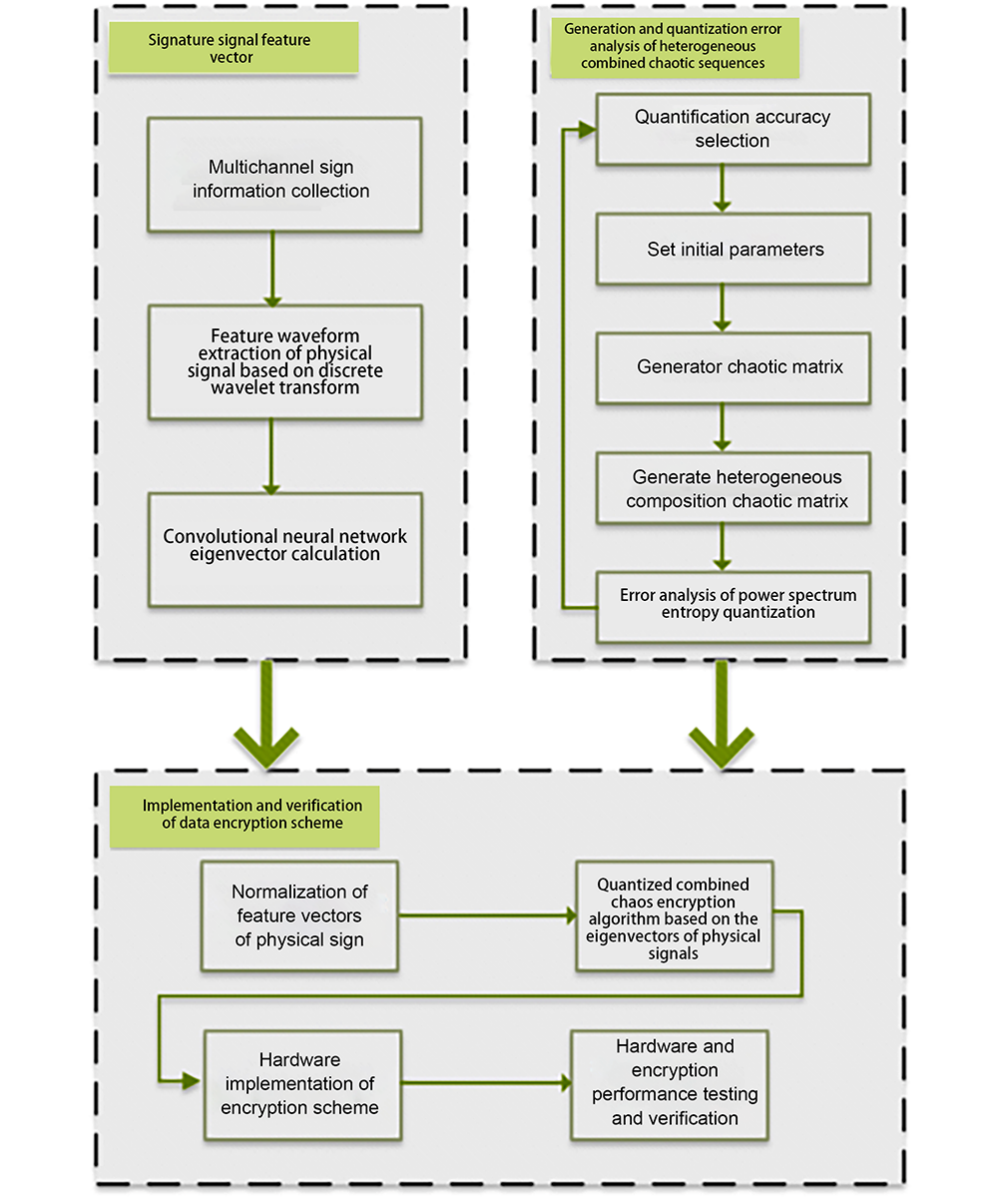
Body area network data encryption method.

The body area network involves sensitive data and hence requires a high-level security system. The limited sensor resources limit the applicability of traditional encryption schemes. As shown in Figure 6, we used the characteristics of physical signals as the initial chaotic values to design a high-strength and sensitive encryption scheme, providing asymmetric encryption with 1 key at a time. The main procedures followed in this study were as follows: (1) calculation of feature vectors for sign signals, precise classification of signal characteristics of physical signs using discrete wavelet transform, and design of multidimensional convolutional neural networks; (2) design and quantification of the algorithm for generating heterogeneous combination chaotic sequences, proposing power spectral entropy as the evaluation criterion, and designing a quantified heterogeneous combination chaotic sequence that met the security requirements of the body area network; and (3) implementation and verification of data encryption scheme, proposing a heterogeneous combination chaotic encryption scheme based on sign signal feature vectors and conducting hardware implementation and performance verification based on the field programmable gate array platform (Figure 6).

### 5G Air-to-Ground Collaborative Rapid Diagnosis and Treatment Technology

#### Development of a Plug-and-Play Highly Reliable 5G Emergency Equipment

**Figure 7:**
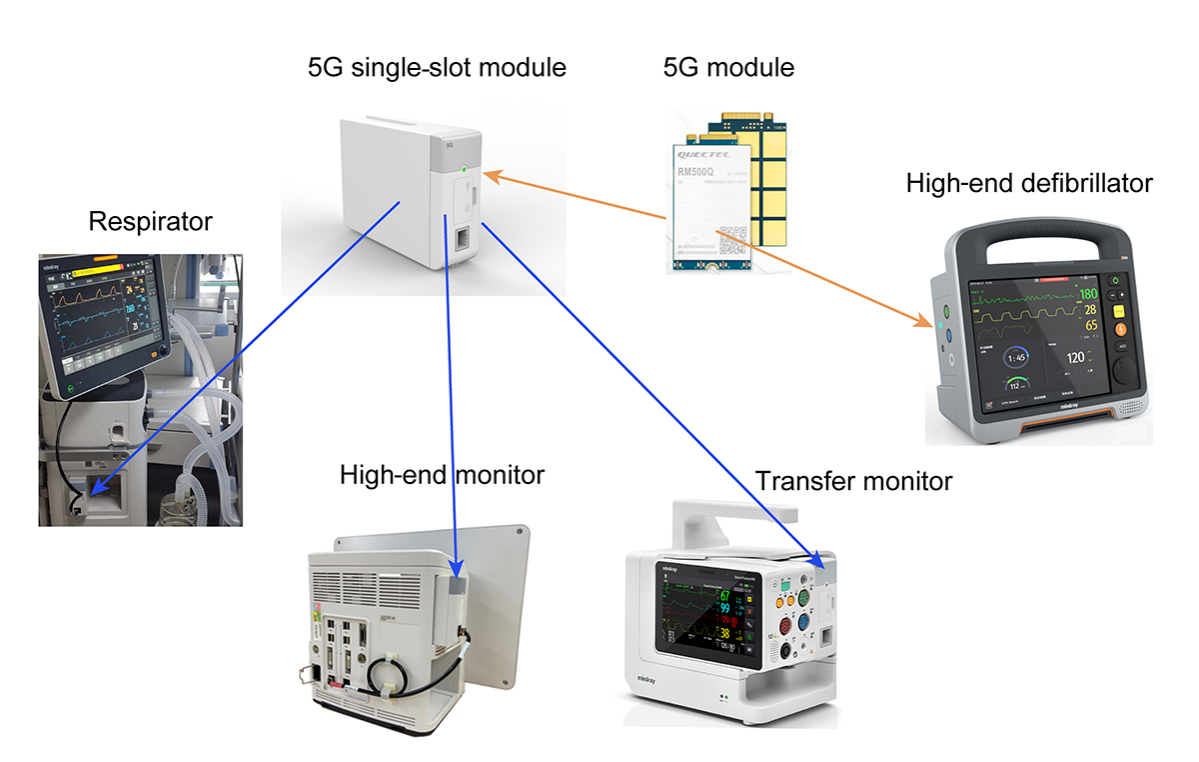
5G first-aid equipment.

The 5G module has high power consumption, insufficient USB power supply capacity for digital medical devices, and unreliable long-term continuous data transmission and communication through a USB module. (1) We developed a 5G single-slot module using the existing 12-V power supply and direct communication between network ports of digital medical equipment. Additionally, we used anti-interference and heat treatment technologies to achieve 5G standardization of devices such as monitors and ventilators. There was no need to change the software and hardware of the digital equipment and special installation. This provided us with “plug-and-play” 5G emergency equipment for use in hospitals (Figure 7). (2) We established highly reliable 5G medical equipment. Our team developed the antenna technology in the form of multiple antennas; coupled devices, antipower technology, and anti-interference technology between equipment; and designed switching performance improvement, network disconnection alarm optimization, and alarm strategy under high-speed movement with highly stable software design technology and wireless parameter tuning. This antenna technology also included switching performance improvement under high-speed movement, network outage alarm optimization, alarm strategy, and other designs, with high reliability of 5G monitors, 5G transport monitors, 5G ventilators, 5G defibrillators, and other equipment. The packet loss rate of the data was within 0.5% at high speeds and edge locations with a reference signal received power of –95 dBm for extended periods, which was much higher in reliability compared with other wireless communication consumer products (packet loss rate of 3%-5%). The 5G rescue equipment developed in this study completed the first domestic air rescue and remote intensive care unit rescue application, playing an essential role in epidemic prevention and control (Figure 7).

#### Establishing an Air-to-Ground Collaborative Information-Sharing Platform

**Figure 8:**
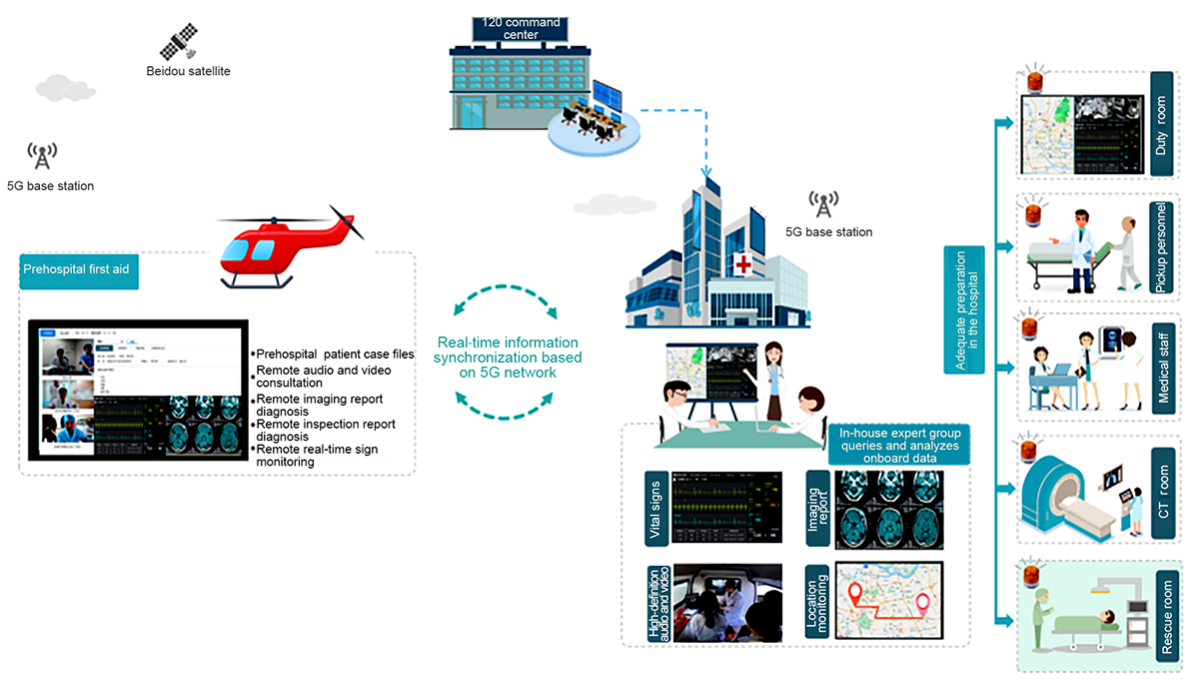
Air-to-ground collaborative information-sharing platform. CT: computed tomography.

A panoramic archive storage and retrieval system was developed for prehospital emergency patients. A panoramic archive of prehospital emergency patients was formed based on the patient health archive, combined with the vital signs collected by 5G emergency equipment, which helped achieve real-time data exchange with the hospital system. We focused on building a standard for emergency rescue data in sky-ground spaces, considering that the panoramic archive of prehospital emergency patients included emergency dispatch and diagnosis and treatment information to support comprehensive and complete access and transmission of cross-institutional rescue information. An air-to-ground emergency rescue information exchange and scheduling system was developed. We used microservice, distributed, and message service technologies to develop an application platform relying on the 5G private network and information security infrastructure, achieving information collaboration covering the entire chain of prehospital first-aid, interhospital transportation, and in-hospital treatment. These technologies supported information exchange and business collaborative applications among cross-regional, cross-institutional, and cross-departmental application systems. By using the Beidou module for satellite positioning and leveraging the high-speed, low-latency characteristics of 5G technology, we can support aviation transportation tracking, injury assessment and management, and remote real-time monitoring and diagnosis of vital signs. It also opened up an air green channel, significantly improving the overall rescue success rate. We developed a remote and multidisciplinary consultation system for sky-ground spaces, established a multichannel collection and transmission of vital signs, and transmitted the archival information and treatment images of patients who are critically ill and outside the hospital to the hospital’s emergency expert group or multidisciplinary consultation on time. This provided technical guidance and full monitoring for the diagnosis and treatment of patients who are critically ill and outside the hospital and improved the efficiency of diagnosis and treatment (Figure 8).

## Our Direct Experience of the Helicopter Aviation Medical Rescue System

### Applications of 5G Technologies in 2 Cases

On August 7, 2022, the aviation rescue drill successfully adopted 5G private network access, achieving advanced functions such as remote video consultation, vital sign data transmission, and the high-precision positioning of Beidou, which transmits the rescue trajectory of the rescue helicopter to the hospital emergency platform in real time through the 5G private network. This drill was novel in testing domestically produced portable extracorporeal membrane oxygenation on a helicopter, laying the technical foundation for practical rescue [10]. These achievements were successfully applied in the first international cross-regional long-distance aviation medical rescue case on November 9, 2022. This case further optimized the application of advanced technologies such as remote video consultation, innovative 5G module equipment, vital sign data transmission, and Beidou high-precision positioning; improved rescue time and efficiency; and shortened transit time [11]. The case validated the low airspace medical rescue 5G network technology, air body area network disease perception technology, and 5G air-to-ground collaborative rapid diagnosis and treatment technology. The entire rescue process seamlessly connected the accident scene via the applications of 5G technologies, medical ambulances or helicopters, and hospital emergency departments, achieving “admission as soon as boarding” and “integration of prehospital and in-hospital diagnosis and treatment,” significantly improving the level of medical rescue.

### Ethical Considerations

The study was conducted in accordance with the principles of the declaration of Helsinki. The study involving human participants was approved by the Ethics Committee of Shenzhen University General Hospital (KYLL-2023019A). All participants provided written informed consent before participating in the study. In this study, the principles of patient privacy protection were strictly followed. Identifying information, including patients’ names, initials, or hospital numbers, was not disclosed.

### Clinical Rescue Effectiveness

#### Improvement in Response Time

The helicopter had a flight distance of 60-600 km and a flight time of 10-136 minutes to cover this distance. In contrast, ambulances typically traveled at speeds of 40-60 km/hour depending on road conditions and distance and covered a distance of 60-600 km in 1-15 hours. Compared to the traditional ambulance, the flight time was less than a quarter of the traditional ambulance time, significantly reducing the rescue response time.

#### Innovative 5G Medical Equipment

On August 7, 2022, the aviation rescue trial first adopted the 5G private network access. On November 9, 2022, the 5G technology assisted the first international cross-regional long-distance aviation medical rescue operation using the novel airworthy 5G module vital sign monitoring equipment jointly developed with Mindray. The 5G emergency equipment could improve treatment efficiency [11,14].

#### Expanding the Rescue Scope

The traditional rescue radius is generally 5-10 km. However, in this study, the helicopter rescue radius was 60-600 km. The helicopter rescue could significantly expand the rescue scope and improve rescue efficiency compared with traditional ambulance vehicles expanding the rescue radius to 600 km.

#### Improving Rescue Effectiveness

A helicopter using 5G technology can transmit real-time high-definition images and videos through high-speed networks, helping doctors conduct remote diagnosis and treatment in the air. Further, 5G technology can also provide high-precision positioning and map navigation functions, helping rescue personnel locate patients more accurately, shortening search and rescue response time, and improving rescue efficiency.

## Technical Application Effect

Previous study showed an improved model of helicopter air-ground cooperative rescue shortened the rescue time by 7.51% and reduced the rescue cost by 4.18% compared with a previous model [15]. Another study of 36 critical cases transferred between hospitals by emergency helicopters showed that the average transfer time was 54.95 (SD 17.89) minutes, and the average transfer distance was 205.74 (SD 74.68) km [16]. Such time for transfer requires airborne medical equipment and rescue techniques. A study used remote mobile medical technology, incorporating Internet of Things technology and a cloud computing system, by deploying information sensing devices on traditional transportation methods, such as cars and helicopters, and connecting these devices to the internet to enable intelligent identification, positioning, tracking, monitoring, and management [17]. This method can realize real-time video monitoring, call and dispatch command of the rescue scene, and remote real-time transmission of information in the car to improve the efficiency of prehospital first aid. The advancement of 5G low-altitude network communication technology was compared and analyzed in domestic and foreign literatures (Table 1) in order to provide a more scientific and effective decision-making basis for future medical rescue.

**Table 1:** Comparison of similar aviation technological parameters at home and abroad.

Technological parameters			Domestic traditional technology		Similar technologies used abroad		Technology used in this study		Comparison results
**Low-altitude 5G network technology**									
	Communications technology	≤150 m, –108 dB, 95% [18]		≤80 m, –120 dB, 95% [19]		≤300 m, –100 dB, 100% [11]		Internationally leading	
	Positioning technology	Foreign GPS high-precision positioning; dynamic positioning accuracy ≤0.1 m [20]		Foreign GPS high-precision positioning; dynamic positioning accuracy ≤0.1 m [21]		China Beidou satellite high-precision positioning; dynamic positioning accuracy ≤1 m [11]		100% localization of technology	
**Air body area network disease awareness technology**									
	Human channel model	Multipose human model, estimation algorithm based on deep learning [22]		Static human body model [23]		A human model of walking and running states		Internationally leading	
	Encryption algorithm information entropy	7.9992 [24]		7.99930 [25]		7.9993		Similar effect	
	Encryption time	0.0834 [26]		1.476551 [25]		0.0385		Internationally leading	
**5G air-to-ground collaborative rapid diagnosis and treatment technology**									
	5G emergency equipment	Does not support 5G direct connection; Wi-Fi wireless access; packet loss rate <2% [27]		Does not support 5G direct connection; Wi-Fi wireless access; packet loss rate <2% [28]		Supports 5G direct connection with a packet loss rate of ≤0.2% [11]		Internationally leading	
	Remote real-time audio and video multidisciplinary consultation technology	None, mainly focused on intercom voice communication [29]		One-on-one audio and video communication [30]		Supports multiparty remote consultation under 1080P high-definition video image transmission [11]		Internationally leading	

## Future Directions

Due to the delayed development of domestic aviation rescue, the application of 5G technology in low-altitude scenarios is still in its infancy, resulting in limited use of 5G technology in aviation rescue operations. Proposed solutions include enhancing the aviation rescue system, promoting the development of 5G applications in low-altitude scenarios, researching 5G solutions suitable for various situations, strengthening coordination within the industry chain, and engaging in international cooperation. 5G network coverage is insufficient; although this study adopted ground tilt coverage technology in the 4.9-GHz frequency band, low-altitude coverage in some remote areas still needs improvement. The solution includes increasing base stations and optimizing their layout, using high-performance equipment and advanced algorithms, improving low-altitude signal transmission, sharing infrastructure, collaborating with civil aviation and meteorological industries for more information support, and considering satellite communication and ground relay as supplementary methods. Currently, 5G networks still have certain stability issues in high-speed mobile scenarios, which may affect the real-time communication performance of helicopter aviation medical rescue. To enhance the stability and communication quality of 5G networks in high-speed mobile scenarios, it is important to explore and implement advanced signal processing technologies and network architectures. Although this study made breakthroughs in 5G key technologies, its global popularization and promotion still need strengthening. To strengthen collaboration with governments, enterprises, and research institutions, the focus is on collectively advancing the development of international standards and specifications for 5G technology. We will accelerate the construction and application of 5G networks. At the same time, it is necessary to increase the publicity of the advantages and application scenarios of 5G technology to improve the awareness and acceptance of 5G technology by the public and enterprises.

## Improvement of Key Technologies

The key 5G technologies for helicopter aviation medical rescue need the following improvements:

- Improvement in the 5G communication coverage range and signal stability: higher frequency band ground tilt coverage technology can be studied to meet a wider range of low-altitude coverage needs, and multiantenna technology and anti-interference technology can be optimized to improve signal coverage range and stability.
- Improvement in personal positioning technology: besides the existing Beidou positioning terminal, hybrid positioning technology that integrates multiple satellite navigation systems (GPS, GLONASS [Global Navigation Satellite System], etc) can be studied to improve positioning accuracy and reliability.
- Optimizing the transmission scheme of the body area network: the near-body channel estimation algorithm based on deep learning and human body posture recognition can be further optimized to adapt to more complex human motion states and improve data transmission efficiency.
- Enhanced body area network data encryption method: more efficient encryption algorithms and protocols can be studied to improve data transmission security based on signal characteristics of physical signs and chaotic mapping.
- Improving 5G emergency equipment: 5G modular devices with higher transmission rates, lower latency, and lower power consumption can be explored to better adapt to various environments and application scenarios.
- Improvement in the functions of emergency platforms: besides real-time audio and video communications, emergency platforms with more intelligent functions can be developed, such as real-time data analysis, intelligent diagnostic advice, and so forth, to improve the effectiveness and efficiency of aerial medical treatment.

The key technologies of 5G also include millimeter wave communication, multiuser multiple-input multiple-output, network slicing, and edge computing. Millimeter wave communication offers higher data transmission speeds, multiuser multiple-input multiple-output technology supports simultaneous connections for more users, network slicing provides tailored network services for various application scenarios, and edge computing reduces data transmission delays while enhancing real-time performance. The 5G key technologies used in helicopter aviation medical rescue can better meet practical application needs through the aforementioned technological improvements and play a significant role in improving medical rescue efficiency and treatment level.

## Conclusions

This viewpoint suggests that 5G technology had significant advantages in terms of improvement in response rescue time, innovative medical equipment, expansion of rescue scope, and improvement in rescue effectiveness. Although existing technologies have shortcomings in terms of network coverage, network stability, and other aspects, the 5G technologies for helicopter aviation medical rescue can better meet practical application needs. We believe that 5G technology can make breakthroughs in terms of rapid response, advanced equipment, and expanding rescue coverage, playing a significant role in improving the efficiency and level of global emergency medical rescue. Furthermore, research and practice on industrial application expansion, intelligent scheduling and management, and data security assurance can be important directions in developing 5G technologies for helicopter aviation medical rescue in the future.

## Data Availability

The data sets generated and analyzed during this study are available from the corresponding author on reasonable request.
